# Hypoxia-Driven Effects in Cancer: Characterization, Mechanisms, and Therapeutic Implications

**DOI:** 10.3390/cells10030678

**Published:** 2021-03-19

**Authors:** Rachel Shi, Chengheng Liao, Qing Zhang

**Affiliations:** Department of Pathology, University of Texas Southwestern Medical Center, Dallas, TX 75390, USA; rachel.shi@utsouthwestern.edu (R.S.); chengheng.liao@utsouthwestern.edu (C.L.)

**Keywords:** hypoxia, metastasis, hypoxia-inducible factors, chemoresistance

## Abstract

Hypoxia, a common feature of solid tumors, greatly hinders the efficacy of conventional cancer treatments such as chemo-, radio-, and immunotherapy. The depletion of oxygen in proliferating and advanced tumors causes an array of genetic, transcriptional, and metabolic adaptations that promote survival, metastasis, and a clinically malignant phenotype. At the nexus of these interconnected pathways are hypoxia-inducible factors (HIFs) which orchestrate transcriptional responses under hypoxia. The following review summarizes current literature regarding effects of hypoxia on DNA repair, metastasis, epithelial-to-mesenchymal transition, the cancer stem cell phenotype, and therapy resistance. We also discuss mechanisms and pathways, such as HIF signaling, mitochondrial dynamics, exosomes, and the unfolded protein response, that contribute to hypoxia-induced phenotypic changes. Finally, novel therapeutics that target the hypoxic tumor microenvironment or interfere with hypoxia-induced pathways are reviewed.

## 1. Introduction

Understanding the mechanisms by which cells sense oxygen and maintain oxygen homeostasis is of pivotal importance for science and medicine. Only in recent decades have breakthrough discoveries of mechanisms for eukaryotic oxygen sensing been made. In response to changing oxygen concentrations, cells maintain intracellular oxygen homeostasis in a myriad of ways such as mRNA reprogramming, metabolic alterations, reoxygenation via angiogenesis, and avoidance of toxic anaerobic by-products [1]. Oxygen levels can affect the stability of enzymes and other proteins which in turn affect transcriptional responses. The discovery of oxygen-sensing mechanisms has not only generated life-saving therapeutic advancements for cancers, cardiovascular conditions, and renal diseases but also earned Drs. Gregg Semenza, William Kaelin Jr., and Peter Ratcliffe the 2019 Nobel Prize in Physiology or Medicine [2].

Despite modest decreases in mortality rates over recent years, cancer continues to be a burdensome national health issue and the second leading cause of death in the U.S. [3]. Solid tumors in particular are associated with poor clinical prognosis because of systemic metastases and local resistance to anti-cancer therapies. Hypoxia, a condition in which oxygen levels are lower than normal physiological oxygenation of tissues (4–9%), is a hallmark of solid tumors and an indicator of poor prognosis in many cancers including prostate, cervix, breast, head and neck cancers [4]. Hypoxia generally ranges from 1–2% O_2_ but depends on the tumor size, stage, initial oxygenation level, and method of oxygen measurement [5,6]. Tumor physiology and distance to tumor microvessels can influence the extent of hypoxia; some areas can be anoxic (0% oxygen) or severely hypoxic (around 0.2% oxygen). 

There are also different types of hypoxia; for instance, chronic or diffusion-limited hypoxia is defined by a continual state of hypoxia because of the inability of oxygen to diffuse to metabolically active cells. Cycling hypoxia (also known as intermittent or transient hypoxia) is characterized by cyclical fluctuations of acute hypoxia and reoxygenation [7]. Intra-tumoral acute hypoxia is typically induced by the temporary absence or restriction of blood supply [8]. Oxygen homeostasis is maintained in normal tissues through a balance of oxygen supply and demand, but in proliferating tumors, oxygen demand exceeds supply, such as through metabolic reprogramming. Consequently, chaotic angiogenic signaling leads to rapid, disorganized blood vessel formation, mediated by vascular endothelial growth factor (VEGF) [7]. 

Responses to hypoxia are predominantly mediated by HIFs, first identified in the 1990s [9,10]. In canonical HIF signaling, hypoxia leads to the stabilization of the labile protein HIF-1α or HIF-2α which complexes with HIF-β (or ARNT), forming heterodimer HIF-1 or HIF-2 that binds to DNA at hypoxia-response elements (HREs) [11]. HIFs subsequently regulate the transcription of genes that affect cell growth, vascularization, metabolism, and oxygen consumption and delivery in a context-dependent manner [12]. The stability of the HIF-α subunits is determined by the hydroxylation of specific prolyl residues (by prolyl hydroxylases (PHDs) 1–3 or EglNs 2, 1, and 3, respectively). HIF-α can also be hydroxylated at an asparaginyl residue (by factor-inhibiting HIF (FIH)). The prolyl and asparaginyl hydroxylations require oxygen and increase the affinity of HIF-α for the von Hippel-Lindau (VHL) protein, a component of an E3 ubiquitin ligase [13]. Under normoxia, VHL polyubiquitinates HIF-α tagging it for 26 s proteasomal degradation [13], whereas under hypoxic conditions, the activity of prolyl hydroxylases is inhibited, and HIF-α is stabilized and able to form the HIF heterodimer which binds to DNA and incites a transcriptional response. There are three HIF isoforms, each with distinct functions, to be discussed in more detail in Section 3.1. Prolyl hydroxylases belong to a larger superfamily of enzymes known as 2-oxoglutarate-dependent dioxygenases (2OGDDs), many of which (e.g., PHDs, JmjC domain-containing enzymes, and ten eleven translocation (TET) enzymes) are oxygen-sensitive and have increasingly been researched in contexts of cancer [14,15].

Research on cancer hypoxia has spanned decades, driven by numerous research groups, and there have been inconsistencies in the in vitro experimental conditions of acute, chronic, and cycling hypoxia induced under [16]. For instance, acute hypoxia has been studied through exposing cells to continuous hypoxia from a few minutes to 72 h [16,17,18]. In addition, the in vitro experimental methods of acute and chronic hypoxia may not recapitulate in vivo complex hypoxic tumor conditions. In vitro and intra-tumoral hypoxia also result in distinct gene expression signatures [19], complicating extrapolations of results from hypoxic culture experiments to in vivo and clinical settings. Due to these discrepancies, there is no clear consensus on the biological consequences of chronic hypoxia, with one study reporting chronic hypoxic cells demonstrating higher invasiveness than cells exposed to acute hypoxia [20] and another reporting that chronic hypoxia leads to regressive changes [21]. These differences may be attributed to the usage of different cell lines and variable cell states at induction of hypoxia. 

With these considerations in mind, connections between hypoxia and pathological indicators such as tumor proliferation, metastasis, invasion, and cancer stem cell-like phenotype continue to be illuminated in emerging research. The dynamic and heterogeneous distributions of hypoxia elicit unique metabolic signatures and transcriptional reprogramming in cells. Many resulting pathways contribute to cancer proliferation and therapeutically relevant phenotypes [7]. The following review will highlight current knowledge on the effects of hypoxia on mutagenesis, DNA repair mechanisms, metastasis, the stem cell phenotype, and resistance to therapies in the context of cancer. In addition, the hypoxia-mediated drivers of cancer malignancy including HIFs and the unfolded protein response (UPR) will be summarized, along with cancer therapeutics that target the oxygen-sensing pathways and cellular adaptations to hypoxia. 

## 2. Effects of Hypoxia on Cancer Molecular and Cellular Characteristics

### 2.1. Mutagenesis and Impaired DNA Repair

**Figure 1:**
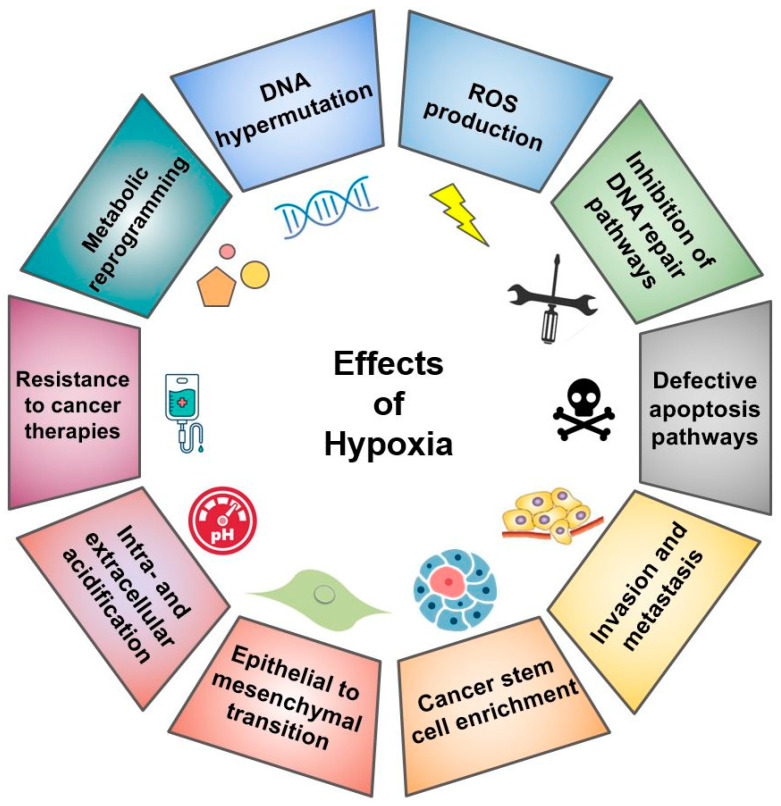
The spectrum of effects of hypoxia on cancer cells. Hypoxia affects cancer cell fate, genetics, metabolism, and clinicopathology. ROS: Reactive oxygen species. DNA: Deoxyribonucleic acid.

Hypoxia generally results in the necrosis of tissues distantly located from host blood vessels and the activation of various pathways that enable surviving cells to transform into malignant, proliferating, clinically relevant tumors [22]. Changes at the genetic level may underlie hypoxic cellular adaptation. A study in 1996 first implicated hypoxia as an environmental factor that is associated with genetic mutations in a tumorigenic cell line [23]. While hypoxia does not directly damage DNA, studies utilizing plasmid reporter systems have shown that hypoxia indirectly induces hypermutation, DNA strand breaks, oxidative base damage, and DNA over-replication (Figure 1) [24,25,26].

The DNA repair response and associated cell cycle and apoptosis signaling is mediated by three major enzymes, ataxia-telangiectasia-mutated kinase (ATM), ataxia telangiectasia and Rad3-related protein (ATR), and DNA-dependent protein kinase (DNA-PK), which belongs to the family of phosphoinositide 3-kinase-related kinases. Whereas ATM and DNA-PK are activated by double-stranded breaks, ATR is activated by replication stress. Hypoxia has been found to promote HIF-1α phosphorylation in an ATM-dependent manner and activate the mechanistic target of rapamycin (mTOR) pathway and subsequent survival and proliferation signals in pediatric solid tumors [27]. Hypoxia and ATM deficiency results in dysregulation of mTORC1, stabilization of p53, and activation of p53-mediated apoptosis [27]. In addition, hypoxia is linked with increased ATM activity and phosphorylation of its substrate Chk2, which causes G2 cell-cycle arrest and protection against apoptosis for colorectal carcinoma cell lines under acute and cycling hypoxia [28]. One consequence of hypoxia is the diminished current of electrons through the electron transport chain (ETC) and an increase in leaking electrons at the ETC, resulting in the production of ROS which damage DNA and set the stage for genomic instability upon reoxygenation [29]. Hypoxia promotes polyploidy, the duplication of the genome without cell division and is associated with genomic instability and defective apoptotic pathways and mitotic checkpoints [30]. Overall, hypoxia results in the differential regulation of genes and enzymes involved in the DNA repair and replication stress response, generally resulting in cancer cells with unchecked cell cycle arrest and decreased apoptosis signaling [21]. 

The inhibition of the two primary DNA double-strand break (DSB) repair pathways—homologous recombination (HR) and non-homologous end joining (NHEJ)—is associated with genetic instability and cancer. DSBs involve the breaking of DNA phosphor-sugar backbones and the dissociation of DNA into two separate molecules and can originate from endogenous ROS production, ionizing radiation, or the activity of DNA alkylating agents [31]. In NHEJ, a Ku70/80 heterodimer binds to the site of the DSB and recruits the DNA-dependent protein kinase catalytic subunit which initiates the repair signaling cascade [32]. In contract, homology-directed repair requires sequence homology to align DSB ends and is mediated by recombinase factors such as RAD51, RAD52, BRCA1, and BRCA2. Under hypoxia, the functional activity of DNA repair enzymes and the expression of mismatch repair genes *MLH1* and *MLH2* and homologous recombination mediators *BRCA1* and *RAD51* is repressed [33,34,35,36], leading to the amplification of damaged DNA and survival suited to a low oxygen microenvironment. ROS production can also result in single-strand breaks, which are repaired with the help of poly(adenosine diphosphate)-ribose polymerase 1 (PARP-1) protein, and inhibition of SSB repair causes an increase in DNA lesions and DSBs. PARP proteins 1–3 have been key cancer targets; in fact, three PARP inhibitors (Olaparib, Rucaparib, and Niraparib) have been approved by the Food & Drug Administration to treat ovarian cancer [37]. For additional discussion on mechanisms of hypoxia and other DNA repair processes such as non-homologous end joining, mismatch repair, and nucleotide excision repair, please refer to the following review [38].

### 2.2. Metastasis

Hypoxia is known to modify gene expression profiles in a fashion that promotes cellular adaptation, survival, and metastasis (Figure 1). Certain physiological environments such as the bone marrow are more hypoxic than others, and surviving metastasis would require adaptation to the unique cellular and molecular niches of distant sites. A metastatic tumor cell must remodel its extracellular matrix (ECM) to detach from the primary tumor site, migrate, and extravasate, before arriving at and proliferating in the metastatic site. Studies show a link between hypoxia and migration, invasion, and metastasis in gastric cancer cells, fibrosarcoma, cervix carcinoma, and melanoma [39,40,41,42]. Oxygen tension values <10 mmHg are associated with an increased mortality rate and risk of metastasis in cancers of the breast, cervix, brain, head, and neck [43]. Hypoxia upregulates genes related to metastasis such as osteopontin (*OPN*), plasminogen activator urokinase receptor (*PLAU*), and lysyl oxidase (*LOX*), which increases invasion and cell adhesion to the extracellular matrix [44,45]. Breast cancer and Waldenström macroglobulinemia cells exposed to acute hypoxia for 24 h in vitro and injected into mice by the tail vein have demonstrated a greater metastatic capacity than equivalent normoxic cells [46,47]. In addition, using an in vivo reporting system to irreversibly identify hypoxic cells, Godet et al. found that breast cancer tumor cells exposed to hypoxia develop an ROS-resistant phenotype and are more prone to survival, invasion, and metastasis [19]. Intermittent but not chronic hypoxia promoted metastasis, clonal diversity, and stem cell marker expression in breast cancer cells [48]. This evidence indicates that hypoxia is closely linked to the metastatic phenotype in cancer. 

### 2.3. The CSC Phenotype

While hematopoietic stem cells have been studied for 60 years or so, cancer stem cells (CSCs) are a relatively recent discovery from the late 1990s. CSCs are defined as a subpopulation of cancer cells resembling embryonic stem cells and possessing sphere-forming potential, an undifferentiated profile, and the capacity to self-renew and proliferate long-term. Dormant and therefore therapy-resistant, CSCs or tumor-initiating cells are often responsible for relapse and can be characterized by the expression of numerous biomarkers such as CD44, CD24, CD29, CD49f, ALDH, CD105, CD133, or CD166, depending on the cancer type [49,50,51,52]. For instance, the most commonly used expression profile for breast cancer stem cells is CD44^+^/CD24^−/low^ and ALDH^+^ [52]. Through flow cytometry and FACS (Fluorescence-activated Cell Sorting) sorting, CSCs have been identified in hypoxic tumor microenvironments (TMEs) of esophageal, renal, gastric, ovarian, and breast cancer [49,50,51,53]. Studies have shown that as low as 100 CD44^+^/CD24^−/low^ cancer cells of the brain, breast, pancreas, lung, and colon can initiate tumors, while greater than 10,000 cells of other non-CSC counterparts were unable to [54,55,56,57]. One study presents evidence that there is turnover and new formation of CSCs in an MMTV-PyMT mouse model of breast cancer [58], that is in line with the plasticity of CSC populations. It is reasonable to speculate that hypoxia and appropriate environmental conditions may promote a bias toward the CSC phenotype. Hypoxia is associated with the loss of differentiated gene expression in hypoxic neuroblastoma and breast cancer cells [59,60] which can promote immortalization and tumor aggressiveness. In addition, hypoxia induces cell cycle arrest, the expression of dormancy genes, and epigenetic modifications such as repressive H3K27me3 found in dormant cells [61]. HIF signaling also upregulates markers of the stem cell phenotype--*SOX9*, *SOX2*, *c-MYC*, *OCT4* and *NANOG*--in 11 cancer cell lines [62]. 

Stem cell-like properties and the epithelial-to-mesenchymal transition (EMT) are intimately connected as embryonic stem cells have been observed to show EMT characteristics, and cells undergoing EMT can acquire a stem cell-like phenotype [63,64]. Although physiologically necessary for embryogenesis, gastrulation, and tissue regeneration, EMT is also considered an early step in cancer metastasis and is associated with pathological organ fibrosis, cancer migration, and chemo- and radio-resistance [65]. In fact, EMT is an important prognostic factor in oral squamous cell carcinoma (OSCC), the most common form of oral cancer [66]. EMT is dependent on transforming growth factor (TGF)-β and the expression of mesenchymal-associated N-cadherin, Vimentin, Snail, and Slug, while inversely related to p53-mediated apoptosis and levels of epithelial-associated E-cadherin (E-cad) [67]. The loss of E-cad is a cornerstone of EMT and is directly regulated by Snail, ZEB, and KLF8, while Twist and Goosecoid are known to indirectly inhibit E-cad [68]. EMT in non-small cell lung cancer (NSCLC) is associated with cell elongation, increased cell motility, and loss of cell–cell adhesion, mediated by HIF1-inducible Snail1 and TWIST [69]. In addition, hypoxia and the subsequent stabilization of HIF-1α promotes EMT in pancreatic cancer cells through NF-κB and TGF-β signaling [70,71]. NF-ĸB upregulates anti-apoptotic gene expression and is likely a factor for chemoresistance in tumors with mutated tumor suppressor KRAS [72,73]. EMT markers are associated with greater resistance to small molecule EGFR kinase inhibitors in cancer cells of the colon, bladder, pancreas, lung, head, and neck with, and restoring E-cad abrogates this resistance [74,75,76,77]. Snail is associated with resistance to paclitaxel, adriamycin, radiation, and dendritic cell immunotherapy in ovarian cancer cells via inhibition of p53-dependent apoptosis [78,79]. In other words, hypoxia induces HIF signaling which upregulates stem cell and EMT markers, promoting associated chemoresistance.

The interactions between cancer stemness and the immune system, described in more detail in another review [80], are another burgeoning field. The CSC phenotype is negatively correlated with natural killer (NK) cells, B cells, and activated CD4^+^ and CD8^+^ T cells in pancreatic cancer and other solid tumors [81,82].

### 2.4. Resistance to Radio- and Chemo-Therapy

Accompanying the complexities of cancer are the multitude of interventions and therapeutics to eradicate it. Nowadays, patients and physicians can consider chemo- and radiotherapy, immunotherapy, and even gene therapy, and treatment management is increasingly more targeted and personalized. However, despite gains and successes in cancer treatment, a cancer cure is far from the horizon, and much work is still needed to further our understanding of the mechanisms of cancer resistance so that treatment can be tailored to maximize cancer eradication in the patient. A minority of patients respond to immunotherapy, and solid tumor heterogeneity complicates the efficacy of chimeric antigen receptor (CAR)-T cell immunotherapy. The most commonly used anticancer treatment is still chemotherapy, but subpopulations of cancer cells, often CSCs, can survive from treatment, proliferate, and become obstacles to disease-free remission [83]. 

Therapeutic successes are often hindered by the hypoxic niche which not only affects tumor itself by reversibly inducing cellular senescence and selecting for a proliferative and metastatic cell phenotype, but also interferes with drug delivery and the cellular uptake of chemotherapeutics. Chemosensitivity depends on the distribution of blood flow within tumors; thus, the irregularity of vascular networks surrounding tumors is correlated with the inconsistent delivery of chemotherapeutic agents. In addition, the altered pH gradients as a result of hypoxia can disrupt the activity of pH-dependent chemotherapeutics (doxorubicin and docetaxel) and DNA alkylating agents, such as temozolomide, chlorambucil, and ifosfamide [84,85]. Hypoxia is already implicated in chemoresistance to doxorubicin, bleomycin, and platinum-based drugs [76,86]. One complication of antiangiogenic drugs, sutinib and bevacizumab, is exacerbation of the hypoxic niche and the subsequent expansion of the CSC population in breast cancer [87]. At the cellular level, hypoxia activates multidrug resistance 1 (*MDR1*), encoding drug efflux pump P-gp, which works to remove intracellular chemotherapeutic drugs, in a HIF-1-dependent manner [88]. The drug action of etoposide which inhibits DNA synthesis is hindered by hypoxia, and liver and breast cancer cells exposed to hypoxia developed more resistance to etoposide than normoxic cells via TMEM45A expression [89]. Overall, hypoxia in solid tumors alters tissue morphology and cellular characteristics in ways that limit the efficacy of many chemotherapeutic agents. 

The efficacy of irradiation treatment owes itself to the mechanism of “oxygen fixation” in which free radicals, the products of ionizing radiation, react with oxygen molecules within cells and irreversibly damage DNA. However, in hypoxic cellular environments, this process occurs less profoundly, and cells can survive. Initial observations in the 1930s found that low oxygen tension and the dearth of oxygen radicals hinders the cytotoxic effects of X- and γ-irradiation [90], and up to three times the radiation dose is needed in hypoxic cells to have similar cytotoxic effects as normoxic cells [91]. Radiosensitivity also depends on cell state (i.e., cell cycle phase), the type of radiation, and oxygen levels in the target tissue region [5]. In vitro and in vivo studies have shown that both short-term and long-term hypoxia result in increased resistance in radiotherapy [92,93,94]. The hypoxia-induced increase in VEGF contributes to angiogenesis which promotes disease recurrence after irradiation [95]. 

**Figure 2:**
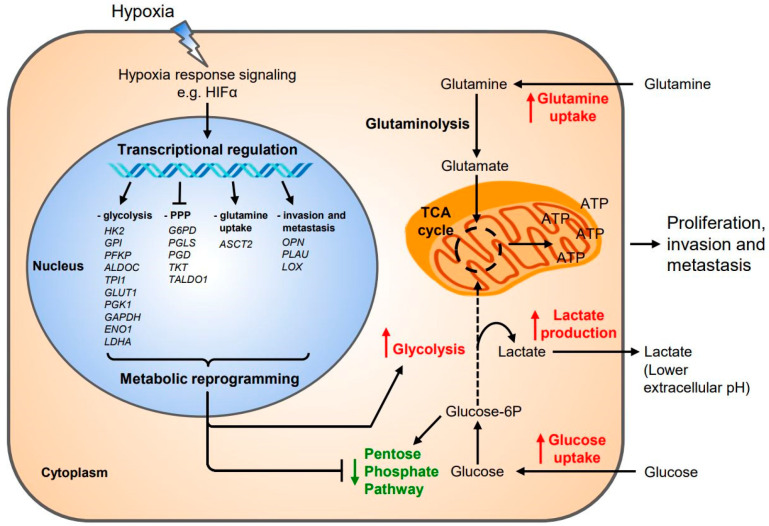
The Transcriptional-Metabolic Interactions under Hypoxia. A variety of genes upregulated transcriptionally under hypoxia affect cancer cell metabolism and behavior. The balance of glycolysis and oxidative phosphorylation along with the amounts of cholesterol, triacylglycerols, and other metabolites can metabolically “prime” a cancer cell to seed at specific organs during metastasis. HK2: Hexokinase 2; GPI: Glucose-6-phosphate isomerase; PFKP: 6-phosphofructokinase platelet type; ALDOC: Aldolase C; GLUT1: Glucose transporter protein type 1; PGK1: Phosphoglycerate kinase 1; GAPDH: Glyceraldehyde-3-phosphate dehydrogenase; ENO1: Enolase 1; LDHA: Lactate dehydrogenase-A; G6PD: Glucose-6-phosphate dehydrogenase; PGLS: 6-phosphogluconolactonase; PGD: 6-phosphogluconate dehydrogenase; TKT: Transketolase; TALDO1: Transaldolase 1; ASCT2: Alanine-serine-cysteine transporter, type-2; OPN: Osteopontin, PLAU: Plasminogen activator urokinase receptor, LOX: Lysyl oxidase; UPR: Unfolded protein response; TCA: Tricarboxylic acid; ATP: Adenosine triphosphate.

In general, hypoxia causes extensive genomic changes and shifts in transcriptional responses which in turn affect metabolic programming and phenotypic expression (Figure 2). Hypoxia is associated with an upregulation in metabolic genes enriched for drug metabolism and pyrimidine and purine synthesis [96]. HIF-1 also activates glycolysis-related genes such as *GLUT1*, *GLUT3*, *PDK1*, *GAPDH*, *ENO1*, and *LDHA* [97]. Hypoxia can also augment the uptake of fatty acids and glutamine, the latter of which can be converted to oncometabolites (e.g., 2-hydroxyglutarate (2-HG), fumarate, and succinate) and be used for ATP synthesis that promotes tumor proliferation [98]. 2-HG can also affect the activity of oxygen-dependent enzymes such as JmjC histone demethylases and DNA demethylases [99]. Shifts to anaerobic glycolysis and lactate production in tumor cells not only lowers extracellular pH which affects drug effectiveness and the immune response [100], but also increases adenosine levels which is known to suppress T cells [101]. In addition, hypoxia decreases the expression of genes related to the pentose phosphate pathway (e.g., *G6PD*, *PGLS*, *PGD*, *TKT*, and *TALDO1*) while upregulating those associated with glycolysis (*HK2*, *PFKP*, *LDHA*) in glioblastoma [102,103]. However, not all cancer cells shift to glycolysis under hypoxia; a bias toward mitochondrial oxidative phosphorylation is observed in certain leukemias, lymphomas, pancreatic ductal adenocarcinoma, endometrial carcinoma, and chemo-resistant melanoma [104]. Breast cancer cells highly metastatic to the brain had high levels of cholesterol, membrane lipids, and metabolites associated with the pentose phosphate pathway and low levels of triacylglycerols, which reflects the brain physiological environment [105]. A connection between hypoxia and the metabolic adaptations in line with the seed-and-soil hypothesis is yet to be investigated. Cancer cell metabolism is dynamic and influences tumor growth, survival, and metastasis in ways unique to different cancers and in vivo contexts; nevertheless, investigating the metabolic effects of hypoxia can potentially reveal novel therapeutic targets and ways to overcome cancer resistance.

A more nascent field explores the role of hypoxia in immunosuppression, immune evasion, and resistance to immunotherapy, which is summarized in another review [106]. Overall, hypoxia elicits complex cellular adaptations comprising genetic and phenotypic modifications which ultimately lead to a malignant phenotype of metastasis and resistance to anti-cancer therapies. A few known mechanisms of these cellular changes will be discussed in the following section.

## 3. Key Drivers of Hypoxia-Mediated Resistance and Metastasis

### 3.1. Hypoxia-Inducible Factors 

HIFs are the predominant mediators of a cell’s metabolic and physiological response to hypoxia and have increasingly been found to influence EMT, metastasis, and chemoresistance. HIF signaling can be activated by the PI3K, AKT, MAPK, and NF-ĸB pathways which can be activated by cytokines such as TNF-α, chemokines, G protein-coupled receptors, Toll-like receptors, and other factors [5]. In triple-negative breast cancer (TNBC), HIF-1 accumulation occurs as a result of glutamate secretion which inhibits the xCT glutamate-cystine antiporter leading to intracellular cysteine depletion [107]. The absence of cysteine under hypoxia subsequently inactivates prolyl hydroxylase EglN1 which usually facilitates HIF-1α degradation in normoxia [107]. Other pathways such as PI3K/AKT signaling, in conjunction with a HIF-1-induced transcriptional response, induced cisplatin chemoresistance and EMT marker expression in hepatocellular carcinoma (HCC) cells [108]. Numerous associations have been highlighted between HIF levels and metastasis, disease recurrence, and poor prognosis in ovarian, breast, thyroid, and lung cancers [109,110,111,112]. HIF-1α expression is associated with reduced disease-free survival and a poorer response to hormone-based therapy in breast cancer [113]. 

In hypoxic conditions, HIFs are stabilized because of the inhibition of oxygen-dependent dioxygenases which modify HIFs under normoxia, enabling its degradation. The stabilization of HIF allows for binding at consensus sequences on DNA and the recruitment of p300, RNA polymerase II, and cofactors so that target genes are transcribed. The downstream effects of increased HIF signaling include regulation of the expression of genes involved in glucose metabolism, erythropoiesis (*EPO*), vascularization (*VEGF*, *SDF1*, *KITL*), tissue remodeling (*LOX*), and wound healing (*TGFA*) [114]. Chromatin immunoprecipitation assays reveal over 1,000 genes regulated by HIF-1 [115], but only a subset of the extensive transactivation, usually 100 genes, is typically observed in a given cell [116].

While there is substantial sequence and structural homology between HIF-1 and HIF-2, they carry out different functions. HIF-1α is the major factor that contributes to target gene transactivation, tumorigenesis, and metastasis in the TNBC cell line, MDA-MB-435 [117]. Both HIF-1α and HIF-2α activate VEGF and form complexes with the c-Myc oncoprotein to regulate transcription [118]. However, HIF-1α inhibits c-Myc activity, whereas HIF-2α potentiates it [119]. In addition, HIF-1α uniquely activates glycolytic enzymes phosphoglycerate kinase (PGK) and aldolase A (ALDA), glucose transporter GLUT1 (or SLC2A), stem cell marker OCT4, the pH regulator, carbonic anyhydrase IX (CA9), and EMT-associated transcription factors Zeb, Snail, and TWIST [120,121,122]. Specifically, CA9 is a prognostic factor, hypoxic indicator, and promising therapeutic target, as inhibiting CA9 can enhance anti-PD-1 and anti-CTLA-4 blockade in melanoma and breast cancer models [123]. In addition, additional targets of HIF-1α include the Wnt and Notch pathways which contribute to EMT, and LOX which activates Snail, represses E-cad, and contributes to chemoresistance in TNBC [121,124,125,126]. Interestingly, HIF-1α but not HIF-2α appears to regulate extracellular acidification in hypoxic tumors while both isoforms contribute to radioresistance in NSCLC [127]. HIF-2 has been identified as the principal oncogenic HIF isoform in clear cell renal cell carcinoma (ccRCC), contributing to a “pseudo-hypoxic” cell state, and has been implicated in pathogenesis, EMT, and angiogenesis in NSCLC [128]. HIF-2 controls cell differentiation and adaptive responses to hypoxia [129,130], along with erythropoiesis [131,132]. The functions of HIF-3α are more elusive due to multiple splicing variants that make functional characterization of the isoform challenging in research. HIF-1α expression can be induced by nitric oxide and ROS [133], but there are also hypoxia-independent mechanisms of regulating HIF by the PI3K/AKT/mTOR pathway, cytokines, epigenetic changes, and lipopolysaccharides, which are not discussed in this review [134].

Recent research has revealed the intersectionality between HIF-1, angiogenic signals, and metastasis. HIF-1 is known to promote vascularization of tumors through the upregulation of VEGF, which in one mechanism, binds to VEGFR2 receptors on bone marrow cells (BMCs), mobilizing the cells to promote angiogenesis [135]. The anthracycline doxorubicin prevents angiogenesis and tumor growth by reducing HIF-1 signaling and the circulation of these BMCs [135]. Similarly in melanoma and lung cancer, colonizing tumor cells produce VEGF-A, LOX, TNF-α, and inflammatory serum amyloid A3 in a HIF-1-dependent manner which leads to the recruitment of bone marrow-derived cells, metastatic niche formation, and remodeling of the extracellular matrix to facilitate invasion [136]. HIF-1α also regulates extracellular matrix metalloproteinase inducer which is induced by hypoxia and promotes metastasis and EMT in esophageal cancer cells [137]. HIF-1 also induces the expression of L1 cell adhesion molecule which allows breast cancer cells to adhere to blood vessel endothelial cells and metastasize to the lung [138]. A study using a mouse model of melanoma found that inactivation of HIF-1α or HIF-2α had no change in tumorigenesis but significantly reduced metastasis, suggesting a specific function of HIF in metastasis [139]. Interestingly, in MDA-MB-231, a human TNBC cell line, VEGF-D is inversely correlated with hypoxia and metastasis to lymph nodes, while platelet-derived growth factor B (PDGF-B) was found to be directly activated by HIF-1 and to facilitate lymphatic metastasis [140]. 

Glycolysis-related HIF-1-transactivated genes might also be a driver in metabolic reprogramming that occurs during metastasis; for instance, HIF-1 activates monocarboxylate transporter 4 (MCT4) expression which promotes the transport of lactate into the ECM, acidifying the TME and favoring pre-metastatic remodeling of ECM [141]. In addition, the metastatic site likely selects for particular metabolic and enzymatic profiles that enable tumor cells to survive. Pyruvate dehydrogenase kinase (PDK1) and glycolytic metabolism facilitated breast metastases to the liver more so than to the bone or lung [142]. 

In addition to the altered milieu of intracellular and extracellular metabolites, hypoxic tumors develop mechanisms that resist the antitumor immune response. The HIF-1-mediated production of TGF-β, VEGF, and CCL28 contributes to immunosuppression in the TME via the recruitment of regulatory T cells (T_regs_), macrophages, and myeloid-derived suppressor cells [143]. T_regs_ are partially responsible for tumor immune tolerance, angiogenesis, and metastasis. Additionally, adenosine production and secretion, associated with an immunosuppressive environment, is increased under hypoxia as a result of the HIF-1α mediated upregulation of CD73 and CD39 [144]. HIF-1 upregulates PD-L1 expression in tumors leading to the overstimulation and exhaustion of T cells, blocking their cytotoxic functions [145]. 

Hypoxia enriches a chemo-resistant CSC population in TNBCs treated with cytotoxic paclitaxel or gemcitabine both in vitro and in vivo through HIF-1α expression [146]. The increase in proportion of breast CSCs was coincided with increased IL-6, IL-8, MDR1, and ROS, and this expression was inhibited with the coadministration of HIF inhibitors digoxin or acriflavine [146]. Both HIF-1α and HIF-2α are known to activate Notch signaling and “stemness” and EMT transcription factors [62,147] which can consequently interact with the Wnt pathway and regulate the stem cell phenotype. Leukemic cells that have adapted to hypoxia exhibit stem cell-like properties along with increased HIF-1α, β-catenin, and glyoxalase-1 activity, an enzyme that detoxifies the harmful glycolytic by-product, methylglyoxal [148]. In this way, hypoxia-induced metabolic, transcriptional, and molecular alterations converge enabling subpopulations of treatment-resistant, stem cell-like cancer cells to survive and contribute to disease relapse.

HIFs drive processes that affect metabolism and cellular behavior, but conversely, EMT and environmental factors such as drug administration can affect cancer metabolism and transcriptional responses. Despite the pervasive influence of HIF pathways on the hypoxic response, some HIF-independent mechanisms for metastasis, EMT, and resistance to therapy may exist in different cancer types and stages. 

### 3.2. Oxoglutarate-Dependent Dioxygenases

2OGDDs are a diverse superfamily of enzymes (e.g., prolyl hydroxylases, JmjC (Jumonji C) domain histone lysine demethylases (KDMs), ten-eleven translocation (TET) DNA hydroxylases, and RNA demethylases such as FTO and ALKBH1-3, 5) that facilitate numerous biological processes, including the HIF-mediated response to hypoxia, ECM formation, DNA and histone modifications, and normal and cancer cell metabolism. 2OGDDs hydroxylate their substrate with the assistance of oxygen, 2-oxoglutarate, and Fe^2+^ and produce succinate in the reaction [15]. In the canonical response to hypoxia, a specific class of 2-OGDDs, prolyl hydroxylase domain proteins, hydroxylate prolyl residues of HIF (Pro402 and Pro564 in HIF-1α; Pro405 and Pro531 in HIF-2α; Pro492 in HIF-3α) which enable the von Hippel-Lindau protein to ubiquitinate HIF-α and tag it for degradation by proteasomes [13]. While dysregulated 2OGDDs have been implicated in many cancers, cancer-related metabolic alterations can also influence 2OGDDs. PHDs (or EglNs) are inhibited by oncometabolites succinate and fumarate, which accumulate under hypoxia, and 2OGDDs can also be targets of HIF signaling. 

Various 2OGDDs have been linked to the development of the CSC phenotype and chemoresistance. PHD2 (EglN1) is inhibited by TGF-β a major factor for EMT and metastasis, resulting in the stabilization of HIF-1 and the enhancement of EMT pathways [149]. In addition, PHD1 (EglN2) inhibition and silencing sensitizes colorectal cancer cells to 5-FU chemotherapy [150]. KDM5A and KDM5B are associated with transcriptomic heterogeneity and therapy resistance in luminal breast cancer and melanoma [151,152]. Loss of KDM6A, a known tumor suppressor, contributes to histone hypermethylation, a phenomenon common in hypoxic tissues, and was found to prevent cellular differentiation under hypoxia, independently from HIF [153]. Likewise, KDM6A and KDM5C are often mutated in ccRCC (3% and 8% of tumors, respectively) [154,155]. KDM6A is also mutated in numerous solid tumors such as bladder cancer, prostate cancer, and breast cancer [156], and depletion of KDM6A led to increased expression of EMT transcription factors, Snail, ZEB1, and ZEB2 [157]. ALKBH5, an m6A RNA demethylase, is responsible for a HIF-1-mediated increase in NANOG expression and the induction of a CSC phenotype in breast cancer [158]. Overall, targeting oncogenic 2OGDDs has potential to not only inhibit tumor growth and CSC features but also enhance existing anticancer therapies. 

### 3.3. The Unfolded Protein Response Pathway

**Figure 3:**
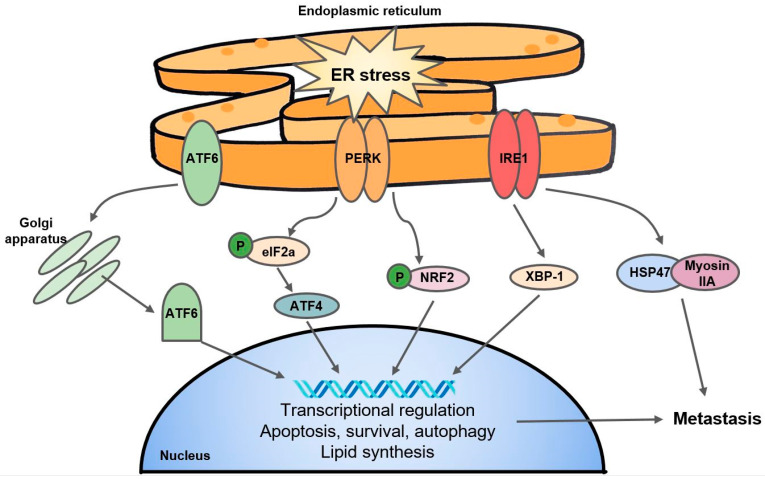
A simple schematic of the UPR. The three major arms of the UPR consist of ATF6, PERK, and IRE1. ATF6: activating transcription factor; PERK: PRKR-like endoplasmic reticulum kinase; IRE1: inositol-requiring enzyme 1; eIF2a: eukaryotic initiation factor 2a; NRF2: Nuclear factor erythroid 2-related factor 2; XBP-1: X-box binding protein 1; HSP47: heat shock protein 47.

Oxygen is a necessary electron acceptor to facilitate disulfide bond formation in protein folding, but under severe hypoxia, impaired protein folding, along with HIF-1-related metabolic switches, mitochondrial stress, or ROS activity can activate the unfolded protein response (UPR) pathway [159]. Although the UPR signaling cascade typically promotes cell survival and adaptation to hypoxia by regulating protein production, degradation, and cell metabolism, it can also induce cell death [160]. The UPR can be characterized by three major endoplasmic reticulum (ER) stress sensors: the PRKR-like endoplasmic reticulum kinase (PERK), inositol-requiring enzyme 1 (IRE1), and the ATF6 pathway (Figure 3) [161]. Together, to mediate ER stress, UPR signals transiently suppress mRNA translation and protein biosynthesis and activate protein degradation and cellular apoptosis [161]. 

Mechanistically, PERK interacts with and phosphorylates eukaryotic initiation factor 2a (eIF2a) which regulates translation of downstream effectors such as activating transcription factor 4 (ATF4). The PERK-ATF4 branch is known to trigger CREB3L1 activity which contributes to metastasis and EMT in breast cancer [162]. The PERK-eIF2a arm of the UPR has been linked to hypoxic cancer cell survival and tolerance to radiotherapy in a colorectal cancer cell line through a mechanism of glutathione synthesis and mitigation of the effects of ROS [163]. Separately, Yoneda et al. demonstrate that IRE1, another UPR transducer, facilitates the interaction of HSP47, a chemical chaperone, with non-muscle myosin IIA, contributing to aggressive metastasis in breast cancer [164]. The expression of XBP-1, another substrate of IRE1, was found to drive TNBC growth and invasiveness, and correlated with hypoxia-mediated HIF-1α gene signatures [165]. Interestingly, the IRE1-XBP-1 axis is also responsible for the downregulation of c-Myc and activation of NK cells which protected against melanoma [166]. In addition, the fact that UPR inhibitors such as 4-PBA and TUD-CA can stall tumor growth and metastasis [167] suggest that the UPR pathway may present as a potential anticancer target. Interestingly, researchers have found that KDM1A inhibitors are able to induce differentiation in glioma stem cells by activating the UPR [168], and ATF4 stability is mediated by PHD3 [169], speaking to the complex and understudied overlap of 2OGDDs with the UPR.

### 3.4. Other Emerging Pathways, from Exosomes to Noncoding RNAs

Exosomes or microvesicles are nanosized vesicles secreted extracellularly that aid in cell–cell communication and sculpting the TME. Able to carry proteins, lipids, microRNAs, or mRNAs, exosomes have been associated with cancer progression, angiogenesis, and EMT. Hypoxia induces the increased release of exosomes in glioblastoma cells along with ovarian, breast, and prostate cancer cells [170,171,172,173]. Hypoxia also influences the composition of molecules within exosomes, such as increasing amounts of triglycerides, metalloproteinases, IL-8, LOX, and heat shock proteins, and inducing the exosomal secretion of miRNAs that induce angiogenesis and stemness [170,174,175,176]. Ovarian cancer cells upregulate the exosomal efflux of cisplatin when treated with the drug, and inhibiting exosome release using Amiloride impaired tumor cell proliferation [173]. Exosomes derived from CSCs promote the survival of immunosuppressive neutrophils, ultimately accelerating colon cancer growth [177]. The secretion of exosomes may in part be mediated by HIF-1 [178,179]. Overall, these data suggest that hypoxia-induced exosomes may contribute to tumorigenesis and chemoresistance. 

Mitochondrial dynamics such as mitochondrial fission and motility influence cell survival, morphology, and ROS homeostasis which may affect EMT and metastasis. For instance, the distribution and motility of mitochondria which is dependent on MIRO1 and MIRO2, Rho-GTPases that regulate mitochondrial movement through anchorage to kinesin or dynein, has been found to influence cancer metastasis [180]. In addition, Dynamin related protein 1 (Drp1) facilitates mitochondrial fission and is upregulated by hypoxia in MDA-MB-231 TNBC cells; silencing of Drp1 reduced mitochondrial fission, ROS production, apoptosis, and migration in TNBC cells [181]. While there is evidence linking Drp1 with stemness in ER-positive breast cancer cells [182], there is a need for additional research to definitely link mitochondrial fission and other dynamics with the CSC phenotype. Previously, our research indicated that PHD1 (EglN2) promotes the binding of peroxisome proliferator-activated receptor-γ coactivator (PGC1α) with NRF1 under hypoxia and subsequently maintains microchondrial biogenesis in breast cancer through inducing transcription of ferridoxin reductase (FDXR) [183]. Recently, protein-tyrosine phosphatase mitochondrial 1 (PTPMT1), an enzyme essential for cardiolipin biosynthesis and mitochondrial membrane integrity, was identified in a genome-wide CRISPR-Cas9 knockout library screening as a crucial survival factor for HCC cells under hypoxia [184].

PTPMT1 regulates cardiolipin synthesis and facilitates the assembly of the ETC complexes which alleviates ROS accumulation during hypoxia. Depletion or pharmacological inhibition of PTPMT1 decreases tumor growth, disrupts the mitochondrial membrane and ETC formation, and reduced metastasis in different cancers [184]. In addition, hypoxia-induced mitochondrial stress is a hallmark of intra-tumoral T cells with persistent antigen stimulation. These exhausted T cells have repressed PGC1α and are less able to mitigate the effects of ROS, suggesting that hypoxia-induced ROS might be connected to T cell exhaustion and dysfunction [185]. By targeting and reversing hypoxia, terminal T cell exhaustion can be prevented, increasing the efficacy of checkpoint blockade immunotherapy [185]. 

The effects of microRNAs (miRNAs) on metastasis, the CSC phenotype, and resistance to antitumor therapies are an understudied field. MiRNAs, while too short to encode proteins themselves, can inhibit the translation of mRNA or facilitate the degradation of target mRNA. With the potential to regulate the expression of a variety of proteins, miRNAs can be tumor suppressive or oncogenic. Hypoxia-mediated miRNA, miR-210, was upregulated in the CSC subpopulation of MCF-7 breast cancer cells, and suppressed E-cadherin and upregulated Snail expression in the breast CSC population [186]. On the other hand, hypoxia-inducible miR-155 repressed homology-directed repair factors such as RAD51 in breast cancer cells and enhanced sensitivity to irradiation [187]. A review paper provides a list of miRNAs that contribute to resistance to chemotherapy agents in many cancers including those of the breast, ovary, stomach, colon, and lung [188]. 

Several studies have pointed out the role of long noncoding RNAs (lncRNAs), noncoding strands of RNA longer than 200 nucleotides, in regulating cancer development. A substantial proportion of single-nucleotide polymorphisms linked to risk in cancers are encoded on lncRNAs, and like miRNAs, lncRNAS can also regulate mRNA translation and degradation [189]. LncRNAs have been found to promote metastasis in OSCC by operating through pathways known to induce EMT such as the AKT, Wnt, and NF-ĸB pathways [190,191,192]. Recently, it was discovered that RAB11B-AS1, a lncRNA transcriptionally induced by HIF-2, promotes angiogenesis and metastasis in TNBC tumors grown in mice through the upregulation of VEGFA and ANGPTL4 [193].

The relationship between circular RNA, defined as single-stranded noncoding RNA that forms a continuous loop via a covalent bond between 3′ and 5′ ends, and cancer is a less studied field. One study reports that circHIPK3, mediated by HIF-2α, contributes to metastasis and invasion in hypoxia-adapted gastric cancer cells through the interaction with miR-653-5p and miR-338-3p and subsequent activation of the AKT pathway [194].

Histone deacetylases (HDACs) are a group of enzymes which modify chromatin structures by removing acetyl groups from lysine residues in histones and transcription factors and can therefore directly contribute to epigenetic and transcriptional alterations during cancer adaptation and survival under hypoxia. HDACs are frequently overexpressed across many cancer types and have been implicated in angiogenesis and cancer proliferation. HDAC1-3 repress miRNA-449 in HCC cells, allowing tumorigenic c-MET to promote growth signals [195]. HDAC6 enables α-tubulin deacetylation in hypoxic conditions, allowing EMT factor, SMAD3, to translocate to the nucleus; HDAC6 inhibitors have been shown to inhibit metastasis in TNBC and angiogenesis in gastric cancer cells by reducing HIF-1α and VEGF levels [196,197]. A recent study reveals a novel role for HDAC6 in glycolysis, and inhibition of HDAC6 not only decreases growth and invasion in TNBC but directly increases acetylation of glycolytic enzymes such as GAPDH, aldolase, and enolase [198].

Another outcome of hypoxia is autophagy, in which cellular stress induces the lysosomal degradation and recycling of proteins and damaged organelles into nutrients to maintain cell functions and promote survival. Together, HIF-1 signaling, hypoxia-mediated metabolic reprogramming, the UPR, and mTOR signaling converge on autophagy and ultimately contribute to tumor proliferation and metastasis [199]. Autophagy can be dependent on or independent of HIF-1 signaling, but it has been shown that HIF-1-mediated upregulation of autophagy genes beclin1 (BECN1) and ATG5 enabled lung tumor immune evasion [200]. In addition, BECN1 was responsible for an impaired natural killer cell-mediated antitumor immune response in breast cancer [201]. 

From miRNAs, lncRNAs, exosomes, and the UPR to the complex transcriptional responses induced by HIFs, hypoxia ignites an avalanche of responses that reconfigure cell metabolism, local tumor immunity and vasculature, and sensitivity to additional stressors such as chemo- or radiotherapy. The co-regulation, convergence, and interdependence of multiple pathways within a hypoxic tumor have great implications in the development and enhancement of anticancer treatments.

## 4. From Mechanisms to Therapeutics 

Cancer therapy resistance remains a pressing medical problem, and 90% of failures in chemotherapy occur as resistant tumors metastasize and invade beyond the primary tumor site [188]. Developing multidrug resistance through drug efflux or detoxification, upregulating DNA repair pathways, and epigenetic reprogramming are a few mechanisms by which cancer cells become resistant [188]. Targeting hypoxia-induced mechanisms of resistance can increase the efficacy of anti-cancer therapeutics. Therapeutics with optimal efficacy must exhibit and maintain targeted lethality to tumors while considering tumor transcriptional and genetic heterogeneity and extrinsic factors such as varied oxygen levels and pH within the TME. Various strategies to overcome resistance and reverse the hypoxic barrier include 1) tumor oxygenation via supplemental oxygen and oxygen transport agents (hemoglobin and fluorocarbons), 2) prodrugs whose antitumor effects are activated under hypoxia, and 3) pharmaceuticals that target hypoxia-induced pathways that lead to hypoxia tolerance and survival [85]. Since hypoxia-based treatments cannot completely cure cancer, these interventions are usually performed in combination with standard cancer therapies to enhance efficacy. 

### 4.1. Drugs That Target the Hypoxic Tumor Microenvironment 

Many chemotherapeutic drugs that are dependent on oxygen for their biological action fail to achieve success in eradicating advanced tumors. One strategy is to reverse tumor hypoxia through oxygen delivery. Tumor oxygenation using oxygen-carrying perfluorocarbon nanodroplets improved the efficacy of radiotherapy and photodynamic therapy in mouse models of breast and colon cancer [202]. Another method to address the hypoxic barrier is to design therapeutics with enhanced or activated cytotoxicity in the hypoxic TME. Originally derived from mitomycin-C which was found to be more cytotoxic in hypoxic conditions [203], hypoxia-activated prodrugs or bioreductive alkylating agents are one class of drugs with chemical signatures (e.g., nitroimidazole groups, azogroups, disulfide bonds, and transition-metal complexes) that can be responsive to reductive hypoxic environments. Such bioreductive prodrugs become “activated” by oxidoreductases and are converted to cytotoxic forms under hypoxia and selectively target poorly oxygenated cells [204]. Theoretically, chemotherapy or radiotherapy would kill less hypoxic regions of tumors, while prodrugs can complement by killing more hypoxic, resistant cancer cells. Mechanistically, prodrugs such as tirapazamine (TPZ) commonly interfere with DNA replication at the replication fork and inhibit cell proliferation [205]; however there are various drawbacks and considerations for using prodrugs. The first generation of prodrugs when used in combination with conventional chemotherapy were often too cytotoxic. In addition, their activation is dependent on the presence of reductases within hypoxic cancer cells which is an understudied field, and different rates of metabolic consumption of the prodrug, dependent on cell density, can affect whether the drug can penetrate all hypoxic region. 

Numerous bioreductive prodrugs have demonstrated preclinical promise [206,207] but fail to demonstrate efficacy in phase II clinical trials [207], possibly because of a physiological distinction between in vitro hypoxic conditions testing the drug and in vivo tumor environments in patients. Specifically, improving stratification of patients based on tumor hypoxia status (i.e., randomizing hypoxic biomarker-enriched or hypoxia-positive patients between treatment groups) would likely optimize the benefits of hypoxia-activated prodrugs and reduce the number of patients required for the clinical trial [208]. The failure of some Phase III trials (e.g., TPZ and TH-302/Evofosfamide) might be attributed to the lack of patient stratification which strongly indicates the need for determining a biomarker-positive threshold in Phase II trials [208]. More granular patient stratification can help distinguish hypoxic tumors that are more likely to be responsive to prodrugs. Overall, there is a need to develop methods to individually characterize the severity of hypoxia and genomic heterogeneity of tumors to better match patients with hypoxia-targeted therapeutics.

### 4.2. Therapies Targeting Hypoxia-Mediated Pathways

Given the complex interactions between hypoxia-induced signaling pathways, targeting a single factor is usually insufficient to halt cancer growth or eradicate tumors. HIF-1α/HIF-2α inhibitors can be categorized as indirect or direct. Indirect HIF inhibitors regulate upstream and downstream effectors in the HIF pathway, while direct inhibitors decrease HIF mRNA expression, protein synthesis, or DNA binding [209]. Numerous HIF inhibitors are undergoing clinical trials such as vorinostat for relapsed lymphomas, PT2385 and PT2977 for ccRCC, and CRLX101 plus bevacizumab for platinum-resistant ovarian, tubal, and primary peritoneal cancer [209]. PT2385 and PT2977 are closely related HIF-2 inhibitors that show promise in targeting a key tumor dependency in ccRCC [210,211]. HIF inhibition also prevents unwanted side effects of irradiation in glioblastoma such as vasculogenesis and disease recurrence [212]. Pharmacological agents that inhibit HIFs (YC-1, 2-methoxyestradiol, anthracyclines) decrease proliferation and metastasis in several types of cancer or can enhance the efficacy of conventional therapies. Treatment with YC-1, for instance, overcomes resistance to gefitinib in NSCLC cells by inducing the degradation of EGFR [213]. 2-methoxyestradiol targets the radioresistant, stem-like cell population in nasopharyngeal carcinoma and downregulates EMT and NF-κB/HIF-1α signaling [214]. Idarubicin, an anthracycline, inhibits metastasis in neuroendocrine tumors by preventing the binding of HIFs to HREs [215]. 

The indirect inhibition strategy involves targeting HIF regulators (including some 2OGDDs) or HIF-mediated transcriptional responses. We have recently published on the therapeutic potential of targeting USP37, a deubiquitinase which reverses the degradation process of HIF-2α in ccRCC [216]. Depletion of USP37 impairs ccRCC growth in 2D and 3D growth assays and in vivo kidney tumorigenesis and lung metastasis [216]; therefore, inhibiting USP37 could be a viable therapeutic approach in VHL-deficient or HIF-2α-dependent tumors. In addition, an inhibitor of NF-κB activity, BAY 11-7082, prevented EMT and reversed resistance to gemcitabine in pancreatic cancer cells [70]. LBH589 (panobinostat), a pan-HDAC inhibitor that indirectly promotes the degradation of HIF-1α, demonstrates antitumor effects in HCC, pancreatic cancer, NSCLC, and glioblastoma [217,218].

2-OG oxygenase inhibitors can not only affect HIF stability as described in Section 3.2 but also alter mitochondrial respiration, cell metabolism, and survival. Particularly, we highlight gamma-butyrobetaine hydroxylase 1 (BBOX1) a 2-OG oxygenase which is known to regulate cellular bioenergetics, calcium balance, and mitochondrial respiration [219]. BBOX1 depletion and pharmacological inhibition results in the degradation of calcium channel protein IP3R3 which subsequently decreases glycolytic pathways, mTORC1 signaling, and TNBC cell growth [219]. Various 2-OG inhibitors and the disease contexts they have been tested in are highlighted below (Table 1). 

Current research has gravitated toward identifying synthetic lethality targets to overcome therapy-resistance and impair cancer growth. We recently identified TBK1 as a synthetic lethal target in VHL-deficient kidney cancer [231], and inhibitors of TBK1 such as BX795, CYT387 481 (momelotinib), and MRT67307 can boost the efficacy of immune checkpoint inhibitors [232]. In addition, the use of veliparib, a drug that targets PARP-1, in combination with RAD51 deficiency, resensitizes hypoxic tumor colon carcinoma cells to radiation [233]. 

Another emerging therapeutic direction is combinatorial treatments, especially those that target hypoxic signaling to enhance immunotherapy. The efficacy of T cell checkpoint blockade immunotherapy in typically unresponsive “cold” tumors such as prostate cancer is enhanced by the combination of a hypoxia-activated prodrug [234]. Recently, Zhou et al. report the use of nanoparticles loaded with sorafenib which reduce tumor hypoxia and trigger the immune response, and when combined with anti-PD-L1 immunotherapy, was able to prevent tumor growth and inhibit metastasis in hepatocellular carcinoma [235]. In addition, vascular normalization through VEGF blockade is an area of interest to assist with the recruitment of cytotoxic T cells to combat tumor resistance [106]. Finally, anti-adenosinergic drugs in combination with PD-1/PD-L1 therapy have reached clinical trials to treat renal cell carcinoma [236], and targeting the immunosuppressive HIF-1-adenosine axis might be a suitable approach to maximize the antitumor response during immunotherapy [237].

**Figure 4:**
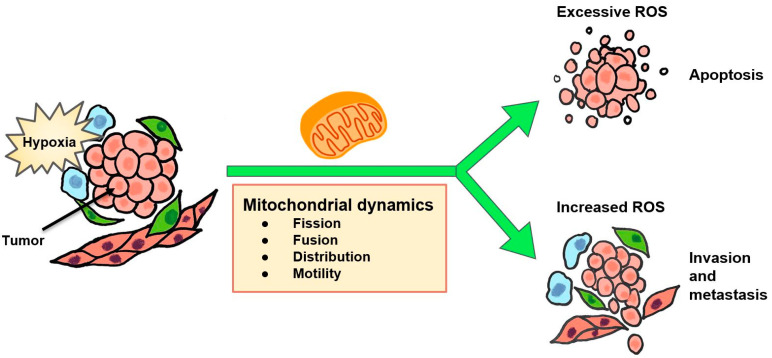
Mitochondrial dynamics may underlie redox homeostasis and the cellular response to hypoxia.

The need to fine-tune therapeutics for individual contexts can be demonstrated by the dual effects of hypoxia-induced oxidative stress (Figure 4); increased ROS is connected to metastasis, but unregulated oxidative stress also leads to cancer cell death [238]. Antioxidants *N*-acetyl-cysteine and vitamin C are thought to be antitumor agents that inhibit HIF-1α activity and genomic instability [133]. However, injecting *N*-acetyl-cysteine to subcutaneous melanoma tumors increased metastasis and survival of melanoma cells to the blood [239], suggesting that oxidative stress could inhibit metastasis in some in vivo contexts. 

As VEGF is upregulated under hypoxia, numerous anti-angiogenic receptor tyrosine kinase (RTK) inhibitors have been developed to block angiogenesis and abrogate tumor growth. However, this is not curative and can exacerbate hypoxia and drive tumor resistance and metastasis. To address this issue, researchers have found that targeting the apelin pathway in combination with sunitinib, an RTK inhibitor, reduced primary tumor growth, local hypoxia, and metastasis in mouse models of breast and lung cancer [240]. In addition, the endogenous antiangiogenic agent semaphorin 3A, when overexpressed, can also enhance the efficacy of antiangiogenic drugs and reduce hypoxia and EMT in a mouse model of pancreatic neuroendocrine cancer [241].

The interconnectedness of metabolic pathways, immune cell interactions, and other aspects of hypoxia-driven cellular reprogramming is a current focus of cancer research and can aid in the identification of novel targets and vulnerabilities. As new mechanisms are elucidated, drug producers are able to consider targeting metabolic reprogramming, pH homeostasis, the DNA damage response, tumor immunity signatures, and other features of cancer cells. As research unravels the crosstalk of pathways that drive tumorigenesis, metastasis, and therapy resistance, it is of increasing importance to develop therapeutics that target multiple pathways in hypoxia-adapted cancer cells and to anticipate interactions and unintended physiological consequences of pharmaceuticals. 

## 5. Conclusions

The numerous effects of hypoxia on cancer progression include changes in DNA repair mechanisms, cell metabolism, and tumoral immunity, along with transcriptional heterogeneity that contribute to the cancer stem cell phenotype, invasion, and resistance to chemo- and radiotherapies. Despite a lack of consensus on in vitro approaches to studying hypoxia, in vivo methods in more physiologically relevant contexts have provided insights to the crosstalk and interactions of numerous proteins and pathways that confer chemo- and radio-resistant, metastatic, malignant, or cancer stem cell-like phenotypes to tumors. Tumor hypoxia presents as a barrier to more effective chemo-, radio-, and immunotherapy. Nevertheless, inhibiting HIF has been a promising therapeutic avenue in disrupting tumor invasion, metastasis, and cancer stem cell enrichment. As cancer treatment increasingly involves combinatorial approaches, future clinical trial designs can include patient stratification based on hypoxic signatures. Understanding mechanisms of hypoxic adaptation can assist in devising more targeted therapies and enhancing the efficacy of current therapeutics. 

## Figures and Tables

**Table 1:** Inhibitors of 2-OG Enzymes.

Inhibitor	Target Protein(s)	Disease Context	References
GSK1278863	PHDs	Anemia of chronic kidney disease	[220]
FG-4592	PHDs	Anemia of chronic kidney disease	[221]
Fisetin	TET1	Renal cancer stem cells	[222]
Compound 10 and 13	KDM5	Lung cancer cell line A549	[223]
NCDM-32B	KDM4	Basal-like breast cancer	[224]
IOX1	KDM3, KDM4	Colorectal cancer and vascular smooth muscle cells in atherosclerosis	[225,226]
GSK2879552	KDM1A	Small cell lung carcinoma	[227]
FB23 and FB23-2	FTO	Acute myeloid leukemia	[228]
Compound 7l	ALKBH3	Prostate cancer cell line DU145	[229]
Indenone derivative Compound 5c	ALKBH3	Lung cancer cell line A549	[230]

## Data Availability

Not applicable.
